# Chemical composition and phytotoxicity of *Eucalyptus parvula* (Hill & Johnson) and *Melaleuca alternifolia* (Cheel) essential oils grown in Tuscany, Italy

**DOI:** 10.3389/fpls.2026.1774330

**Published:** 2026-03-16

**Authors:** Clarissa Clemente, Silvia Tavarini, Guido Flamini, Luciana G. Angelini

**Affiliations:** 1 Department of Agriculture, Food and Environment, University of Pisa, Pisa, Italy; 2 Interdepartmental Research Center “Nutraceuticals and Food for Health”, University of Pisa, Pisa, Italy; 3 Department of Pharmacy, University of Pisa, Pisa, Italy

**Keywords:** chemodiversity, essential oils, eucalyptus oil, Mediterranean cultivation, phytotoxicity, tea tree oil, VOCs

## Abstract

The intensification of global agricultural production to meet growing food demand has led to the widespread and often excessive use of synthetic pesticides, raising significant environmental and health concerns. In this context, plant-derived essential oils represent promising natural alternatives. However, the potential of many species remains largely unexplored, particularly regarding their chemical diversity under non-native cultivation contexts. At this regard, our study focused on the chemical characterization of essential oils (EOs) - in terms of yield, composition, and related volatile organic compounds (VOCs) - and on the potential phytotoxic activity of *Eucalyptus parvula* (Hill & Johnson) and *Melaleuca alternifolia* (Cheel) grown in Tuscany (Italy). To this end, in M. alternifolia, leaves and fruits collected from 3-and 4-year-old plants were analyzed to investigate age-and organ-related chemical variability, whereas in *E. parvula* leaf material harvested in March and July was used to evaluate seasonal effects on chemical composition. Phytotoxic activity was also evaluated through germination bioassays performed on several weed species, including *L. perenne* (L.), *S. marianum* (L.) Gaertn., *R. crispus* (L.), and *P. hieracioides* (L.). Our results revealed that melaleuca EO yield and composition varied with both plant age and organ, with leaves of 4-year-old plants exhibiting higher yields and a greater contribution of oxygenated monoterpenes, mainly 1,8-cineole, whereas fruits had lower yields and a distinct profile enriched in sesquiterpene hydrocarbons. VOC profiles followed similar age-and organ-related trends, and were dominated by monoterpene hydrocarbons such as terpinolene, γ-terpinene, α-terpinene, and 1, 8-cineole. Eucalyptus EO yield showed seasonal variation, while both EO and VOC profiles were dominated by 1,8-cineole with seasonal effects mainly influencing minor constituents. Phytotoxicity assays revealed a dose–dependent response, with increasing percentage inhibition of germination and radicle length as EO doses increased. Although both EOs showed marked inhibitory effects, sensitivity was highly species-specific, as evidenced by differences in IC_50_ values. Tea tree EO exerted a stronger phytotoxic effect than eucalyptus EO, inducing higher levels of inhibition at lower doses. So, this study provides new insight on *E. parvula* and *M. alternifolia* cultivated under Mediterranean conditions, highlighting their complex chemical profiles and potential use as bioherbicidal agents.

## 1 Introduction

Essential oils (EO) and their volatile organic constituents (VOCs) are key components of plant secondary metabolism produced by glandular trichomes and other secretory structures, mainly distributed on the surface of plant organs. They mediate important ecological and physiological roles in plants, including defense against pathogens and herbivores, plant interactions, stress signaling, and pollinator attraction (Guenther, 2013; Rehman et al., 2016; Sharifi‐Rad et al., 2017b; Ben Miri, 2025). In the European Union, Directive 2009/128/EC (http://data.europa.eu/eli/dir/2009/128/oj) and related policies encourage integrated pest management and the reduced reliance on synthetic active ingredients, motivating the development of botanically based products. EOs are attractive candidates because they comprise complex mixtures of bioactive terpenoids that can act through multiple and partially overlapping modes of action, often with synergistic interactions that enhance overall antimicrobial, insecticidal, and phytotoxic efficacy (Borotová et al., 2022). In recent years, EOs are becoming progressively more significant worldwide as bioactive, biodegradable, and multifunctional natural products, with applications in food, pharmaceuticals, and cosmetics, as well as in the agricultural sector, where they are increasingly appreciated as environmentally friendly alternatives to synthetic pesticides (Pavela et al., 2020; Bellache et al., 2022; Gupta et al., 2023; Meryani et al., 2024; Swathika et al., 2025).

The EO chemical composition consists of two main groups of chemicals. The first includes terpenoid compounds, primarily monoterpenes and sesquiterpenes, which are the main constituents of most essential oils. The second group includes non-terpenoid compounds, such as phenylpropanoids, short-chain aliphatic structures, nitrogen-containing compounds, and sulfur-containing compounds (Fotsing and Kezetas, 2020). Monoterpenes and sesquiterpenes are widely recognized as key drivers of the phytotoxic activity of EOs, often acting through synergistic interactions that enhance their overall biological efficacy (Raveau et al., 2020). Therefore, this synergistic mode of action underlies the interest in EOs as promising sources for the development of new bio-based products (Vaou et al., 2022). Among the most commercially and biologically important EO-producing plants, *Eucalyptus* and *Melaleuca* spp., belonging to the Myrtaceae family, occupy a prominent place. These species are native to southeastern Australia and have been used for centuries by Aboriginal communities for their medicinal purposes. To date, they play a central role in the global medicinal plant market due to their well-documented pharmaceutical, antimicrobial, insecticidal, and phytotoxic properties (Carson et al., 2006; Vecchio et al., 2016, Sharifi-Rad et al., 2017a; Borotová et al., 2022; Dziągwa-Becker and Oleszek, 2024; Oder et al., 2024; Shiekh et al., 2025). One of the most widely known eucalyptus species is *Eucalyptus globulus* (Labill.) which produces EOs predominantly rich in 1,8-cineole, a compound with strong antimicrobial, expectorant, and phytotoxic properties (Čmiková et al., 2023; Yip et al., 2024). In contrast, *Melaleuca alternifolia* is valued worldwide for its tea tree oil, typically rich in terpinen-4-ol, γ-terpinene, terpinolene, and 1,8-cineole and other related monoterpenes with documented biological activities such as antibacterial, antifungal, antiviral, acaricidal, insecticidal, and phytotoxic effects (Borotová et al., 2022).

Outside their native‐range habitat, *Eucalyptus* and *Melaleuca* spp. demonstrated good adaptability in non-native regions, and are now cultivated in several countries where different climatic conditions characterized by mild winters, summer drought, and high irradiance can significantly influence EO biosynthesis and VOC emission (Benelli et al., 2013; Silva-Pando and Pino-Pérez, 2016; Tomé et al., 2021; Ter Huurne et al., 2023; Nyssen et al., 2024). In the Mediterranean environments, particularly in Italy, *Eucalyptus* spp. are already widely cultivated for various purposes, including forestry, windbreaks creation, ornamental use and biomass production. Among the less studied species, *E. parvula* is attracting interest in Europe due to its adaptability to colder climates, its rapid growth, and its potential for ornamental and biomass production (Ieri et al., 2019). *M. alternifolia* has been instead introduced for experimental/small-scale cultivation mainly for EO production, although their ecological impacts and naturalization dynamics must also be considered (Benelli et al., 2013; Sgroi et al., 2015; Badalamenti et al., 2018; Palmieri et al., 2020; Tamantini et al., 2025). However, despite their expanding cultivation, little is known about their chemical features when grown in Mediterranean climates, especially in terms of EO yield, chemo-diversity, and seasonally related organ-specific variations. At this regard, it is increasingly evident that EO profiles are not static, but rather dynamic expressions of plant metabolism that respond to various environmental factors such as light, temperature, drought, salinity, seasonal variations, crop water availability, phenological stage as well as harvesting time and the specific plant organ considered (Gosztola et al., 2010; Guenther, 2013; Moghaddam and Mehdizadeh, 2017; Das and Prakash, 2024). Studies conducted on various Myrtaceae species have shown that leaves, young stems, and fruits can substantially differ in their chemical composition, not only in terms of the relative contribution of monoterpenes and sesquiterpenes, but also in the abundance of minor constituents, which can significantly influence the bioactivity (Heikal, 2017; Silveira et al., 2022). Therefore, it is important to investigate the variability of EOs and VOCs when these non-native species are introduced into new environments, in order to understand any changes in their chemical composition that may impact their biological activity and potential industrial applications (Ben Rabeh et al., 2025; Yip et al., 2024). Furthermore, given the growing need for renewable and environmentally friendly resources, the exploration of EO-producing species grown in Mediterranean areas also aligns with emerging research objectives aimed at establishing local supply chains, promoting biodiversity-based production systems, and developing biopesticides with reduced environmental impact (Assadpour et al., 2024; Zanellato et al., 2009). Despite their economic interest and the broad spectrum of recognized biological activities, information on the cultivation of *E. parvula* and *M. alternifolia* in Mediterranean regions is still limited, particularly on how EO yield and VOC composition vary in response to plant age, plant organ, and seasonal variations. Understanding these factors is essential to assess the feasibility of establishing local supply chains for the production of high-value EOs. In this context, our study provided the first assessment of the chemical composition and phytotoxic activity of essential oils and VOCs spontaneously emitted by *E. parvula* and *M. alternifolia* grown in Tuscany, Italy. Specifically, we assessed how (i) plant organ and plant age in *M. alternifolia* and (ii) harvest time in *E. parvula* influenced EO yield and composition, as well as VOC profiles. Thus, by integrating EO analyses, VOC profiles, and phytotoxic bioassays, our study aimed to clarify the metabolic responses of these two Australian species to Mediterranean environmental conditions and to assess their potential integration into sustainable agro-industrial supply chains, as well as their potential use in formulations for bioherbicidal applications.

## 2 Materials and methods

### 2.1 Plant material

Tea tree material was obtained from seeds of an accession supplied by Sand Mountain Herbs (North Alabama, USA). Seed germination was performed according to the International Seed Testing Association (ISTA, 2015) at the Seed Research and Analysis Laboratory (LaRAS) of the Department of Agriculture, Food and Environment (DAFE, University of Pisa). Seven days after sowing, germinated seeds were placed in plug trays filled with peat into a cold greenhouse (temperature: 20± 2 °C) until reaching a suitable size for field establishment. Then, the tea tree seedlings were transplanted into the experimental field at the Tuscany Region Agricultural Center (TeReTo), located at Alberese (ALB, Grosseto province, 42°41′38″, 10°08′29″), in southern Tuscany, at the end of the summer 2020 and leaves and fruits from three-and four-year-old plants were subsequently collected for quali-quantitative analyses. Before transplanting, soil physical and chemical properties at a 30 cm depth were evaluated. The soil showed a sandy-loam texture according to USDA classification with a pH slightly alkaline, a medium level of total nitrogen (1.0%) and organic matter (1.5%). The climate of the area is typically Mediterranean, characterized by mild winters and hot summers. Average monthly temperatures ranged from 6-8 °C in winter to 24-26 °C in summer, with maximum temperatures exceeding 30 °C in July and August. Rainfall is unevenly distributed throughout the year, with the highest amounts recorded in autumn and late winter, particularly between October and December.

Eucalyptus material was obtained by sampling leaves from two-year-old plants cultivated in the littoral area of Versilia (North Tuscany, Italy, 43°52′25″, 10°19′43″) by a commercial farm specialized in the production of green fronds for ornamental purposes. Several species of eucalyptus are grown by organic practices, accredited according to the UNI EN 45011 standard, and the soil supporting their cultivation is of medium-textured with a clayey tendency. The area has a typically Mediterranean climate, characterized by mild winters and hot summers. Average monthly maximum temperatures reach approximately 25-28 °C in July-August, while average monthly minimum temperatures range between 5-8 °C in winter. Rainfall are concentrated mainly in autumn and late winter, with monthly peaks often exceeding 100 mm, while the summer months are generally dry with almost no precipitation. During ornamental frond production, approximately 50% of the harvested biomass is discarded as waste. Leaf material derived from these discarded cut branches was collected and subjected to quali-quantitative analyses.

### 2.2 Extraction and chemical characterization of *Melaleuca* and *Eucalyptus* essential oils

To obtain the EOs, hydrodistillation was performed for 2 h in a Clevenger-type apparatus. For each experimental condition (plant organ, plant age, or harvest time), three independent biological replicates were analyzed, each consisting of 100 g of aerial parts collected from three different individual tea tree and eucalyptus plants. The extracted oils were then conserved at 4 °C and maintained far from any light sources until analyses.

The hydro-distilled essential oils were diluted to 5% in HPLC-grade *n*-hexane and injected into GC–MS equipment. Gas chromatography-electron impact mass spectrometry (GC–EIMS) analyses were performed using an Agilent 7890B gas chromatograph equipped with a HP-5 capillary column (30 m × 0.25 mm; coating thickness 0.25 µm) and an Agilent 5977B quadrupole mass detector (Agilent, Palo Alto, CA, USA). Specifically, the following samples were analyzed using GC-EIMS:

- Essential oils from hydrodistillation of leaves and fruits collected from three and four-year-old melaleuca plants (4YOP and 3YOP);

- Essential oils from hydrodistillation of leaves collected in March and July from 2-year-old eucalyptus plants.

The analytical conditions were the following: injector temperature at 220 °C; transfer line temperature at 240 °C; oven temperature rising from 60 °C to 240 °C at 3 °C/min; carrier gas helium at 1 mL/min. The injection volume was 1 µL, with a split ratio of 1:30. The acquisition parameters were: full scan; scan range: 30–300 m/z; scan time: 1.0 sec. The peak identification relied on a comparison between the retention times with those of the authentic samples, comparing their linear retention indices relative to the series of *n*-hydrocarbons (C8-C27) and a computer matching against commercial (NIST 14 and ADAMS 2007) and laboratory-developed mass spectra libraries built up from pure substances or mixtures of known composition and MS literature data (Stenhagen et al., 1974; Masada, 1976; Jennings and Shibamoto, 1980; Swigar and Silverstein, 1981; Davies, 1990; Adams, 1995). The essential oils were stored into a refrigerator at 4 °C until analysis.

### 2.3 HS-SPME/GC-MS analysis of volatile organic compounds emitted by *Melaleuca* and *Eucalyptus*

A Supelco SPME device coated with polydimethylsiloxane (100 μm; Sigma-Aldrich) was used. The analysis was performed on three independent biological replicates for each experimental condition (organ, plant age, or harvest time), corresponding to samples collected from three different individual tea tree and eucalyptus plants. For tea tree, 3 g of leaves and 1 g of fruits from 3- and 4-year-old plants were analyzed, while for eucalyptus, 2 g of leaves were used deriving from March and July harvest. Each sample was immediately placed in a 50-ml glass conical flask, allowed to equilibrate for 20 minutes, and then exposed to headspace for 15 minutes at room temperature. Once sampling was finished, the fiber was withdrawn into the needle and transferred to the injection port of the GC–MS system, operating under the same conditions, as described above, except for the splitless injection mode and the injector temperature set to 250 °C. A blank run of the fiber was carried out between sample analyses to check for possible carryover effects and to ensure the absence of residual compounds. VOC results are expressed as relative abundance (% peak area).

### 2.4 Evaluation of the phytotoxic activity of essential oils against weed species

The EOs of *M. alternifolia* and *E. parvula* were evaluated for their potential phytotoxic effects on several widespread weed species, including *Lolium perenne* (L.), *Silybum marianum* (L.) Gaertn, *Rumex crispus* (L.), and *Picris hieracioides* (L.). These species are widely distributed across temperate agroecosystems and are known to cause significant yield losses through competition for water and nutrients, interference with crop establishment, and, in some cases, allelopathic effects, as consistently reported in scientific studies on weed management. Lettuce (*Lactuca sativa* L.) and radish (*Raphanus sativus* L.), kindly provided by ViviVerde Coop Italia, were included as standard reference species commonly used in phytotoxicity bioassays due to their rapid and uniform germination rate. For each bioassay, 50 seeds were placed in 9-cm Petri dishes lined with filter paper moistened with 4.5 mL of deionized water and exposed to increasing concentrations of tea tree and eucalyptus essential oils (10, 20, 60, 100, 150 and 200 µL), alongside an untreated control. Each volume of EO was placed in a small container located inside the Petri dish to ensure that seeds were exposed exclusively to the volatile fraction without direct contact with the essential oil. Therefore, in order to minimize evaporation, the Petri dishes were sealed and no additional water was added during the experiment. So, the experimental design included six concentrations of each EO (treated samples) and distilled water (control samples) x 6 target species × 3 replicates (Petri dishes).

Depending on the standard reference/weed species, petri dishes were incubated either at a constant temperature of 20 °C or under alternating temperature conditions (20/30 °C with 16:8h light/dark), following the recommendations of the International Seed Testing Association (ISTA, 2015). Incubation was carried out in a climatic chamber equipped with cool white fluorescent lamps (Osram L18 W/20), providing a light intensity of 10 µmol photons s⁻¹ m⁻². The germination was monitored every 2 days, and final counts for each species were performed according to ISTA (2015).

Germination percentage (G %), mean of germination time (MGT), and germination inhibition (GI), were calculated according to following equations:

- G (%) = S_NG_/S_NO_ × 100, where S_NG_ is the number of germinated seeds and S_NO_ is the total number of seeds in the bioassay, respectively.
- MGT = Σ (n × d)/N, where n is the number of germinated seeds per day; d is the number of days needed for germination, and N is the total number of germinated seeds.
- GI (%) = {[GC−GT)]/GC} × 100, where GC is the germination percentage in the control treatment; GT is the germination percentage in each treatment.

Additionally, radicle length (cm) was measured on ten emerging seedlings for each replicate, for a total of thirty seedlings analyzed and the percentage inhibition of radicle length (RLI) was also calculated using the following equation:

- RLI (%) = (Lc − Lt)/Lc × 100, where Lc is the mean radicle length of the control seedlings (cm) and Lt is the mean radicle length of the treated seedlings (cm).

### 2.5 Statistical analysis

Data were subjected to statistical analysis using GraphPad Prism v. 8.0.2 (GraphPad Software, Inc., La Jolla, CA, USA). Student’s *t*-test was used for comparisons between two independent groups.

For datasets involving plant age (3- and 4-year-old melaleuca plants) and plant organ (leaves and fruits), a two-way ANOVA was applied to evaluate the main effects of each factor and their interaction on EO yield. Furthermore, one-way ANOVA was performed to assess the effects of different concentrations (10, 20, 60, 100, 150, and 200 µL) of tea tree and eucalyptus EOs on seed germination percentage, radicle length, and mean germination time (MGT) of each tested species, in comparison with the untreated control. Differences among means were assessed using the Least Significant Difference (LSD) test, and statistical significance was accepted at *p* ≤ 0.05. Moreover, dose–response curves for inhibition percentage of seed germination and radicle length were fitted by non-linear regression using a variable-slope four-parameter logistic (4PL) model to calculate IC_50_ values. The goodness of fit of the models was evaluated using the coefficient of determination (R²). Principal Component Analysis (PCA) and Hierarchical Cluster Analysis (HCA) were also performed to better visualize the variability of chemical profiles and distinguish the effects of plant age, plant organ, or harvest time depending on species tested, using R Statistical Software (RStudio v1.4.1106, Boston, MA). The PCA was carried out on the correlation matrix with the goal of reducing the dimensionality of the multivariate matrix data (6 samples x 13 variables) whilst preserving most of the variance. Prior to Principal Component Analysis (PCA), compounds with relative abundance < 1% were excluded to reduce noise and avoid over-weighting rare variables in the PCA. Furthermore, means of all samples for each variable have been used and subsequently centered and scaled. The number of principal components (PCs) was identified according to different criteria: the Kaiser–Guttman criterion (eigenvalues > 1), the percentage of variance explained cumulatively, and a scree and elbow plot. Variable weights/loadings were examined to identify the variables that contributed the most to each selected PC. The HCA was conducted on the normalized average values with Ward’s algorithm, using Euclidean distances as a measure of (dis)similarity among the samples. Before applying the hierarchical clustering method, to assess whether the data were clusterable, the Hopkins statistics (H) was used; a 0.69 H value was obtained, indicating a good propensity of our data to form clusters. Values of H close to 0.5 indicate a random spatial distribution, whereas values closer to 1 indicate a stronger cluster tendency (Banerjee and Dave, 2004; Lawson and Jurs, 1990). The result was a hierarchically clustered heatmap performed on the standardized data; this is also called false-color image, in which the data values ​​were transformed into a color scale, showing the similarity among groups based on essential oil chemical composition.

## 3 Results

### 3.1 EO yield of *Melaleuca alternifolia* and *Eucalyptus parvula*

**Figure 1:**
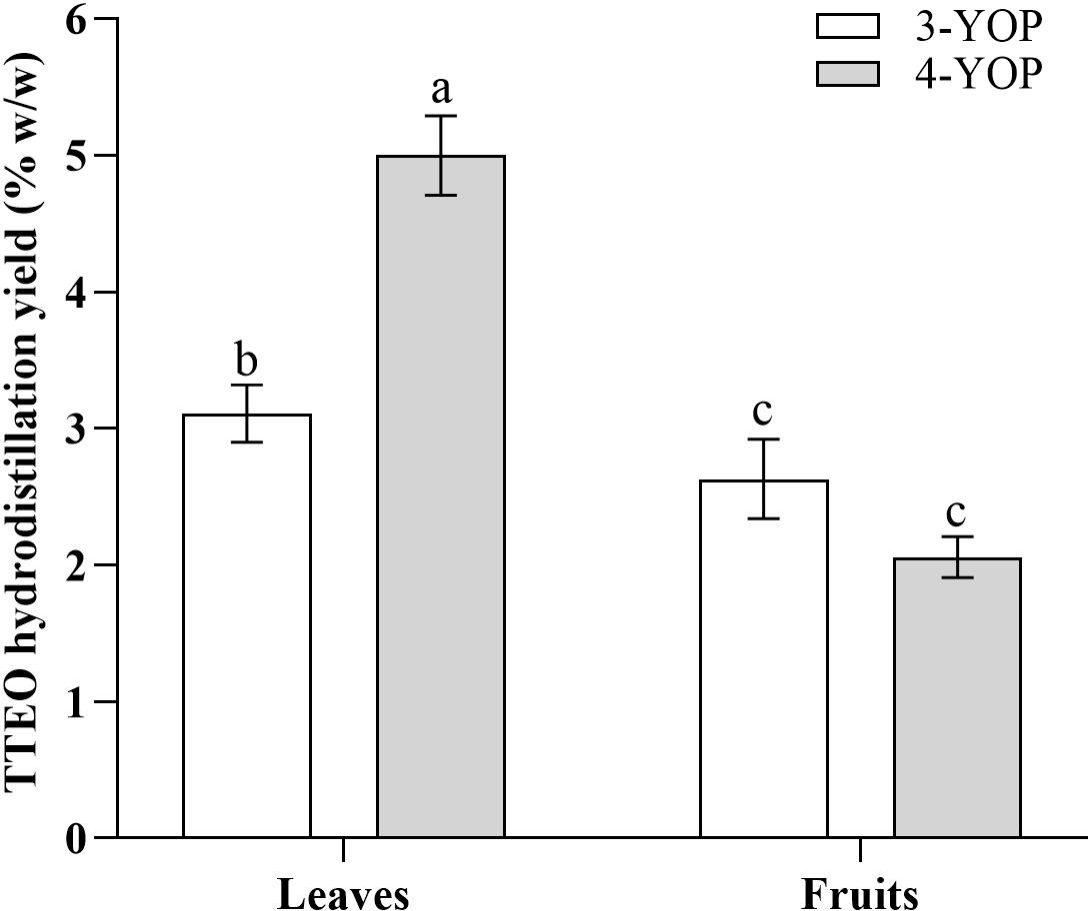
Effect of plant age × plant organ interaction (PA × PO) on TTEO hydrodistillation yield (% w/w). Means followed by different letters are statistically different at *p* ≤ 0.05 according to LSD Fisher’s test. 3YOP = 3-year- old melaleuca plants; 4YOP = 4-year- old melaleuca plants.

**Figure 2:**
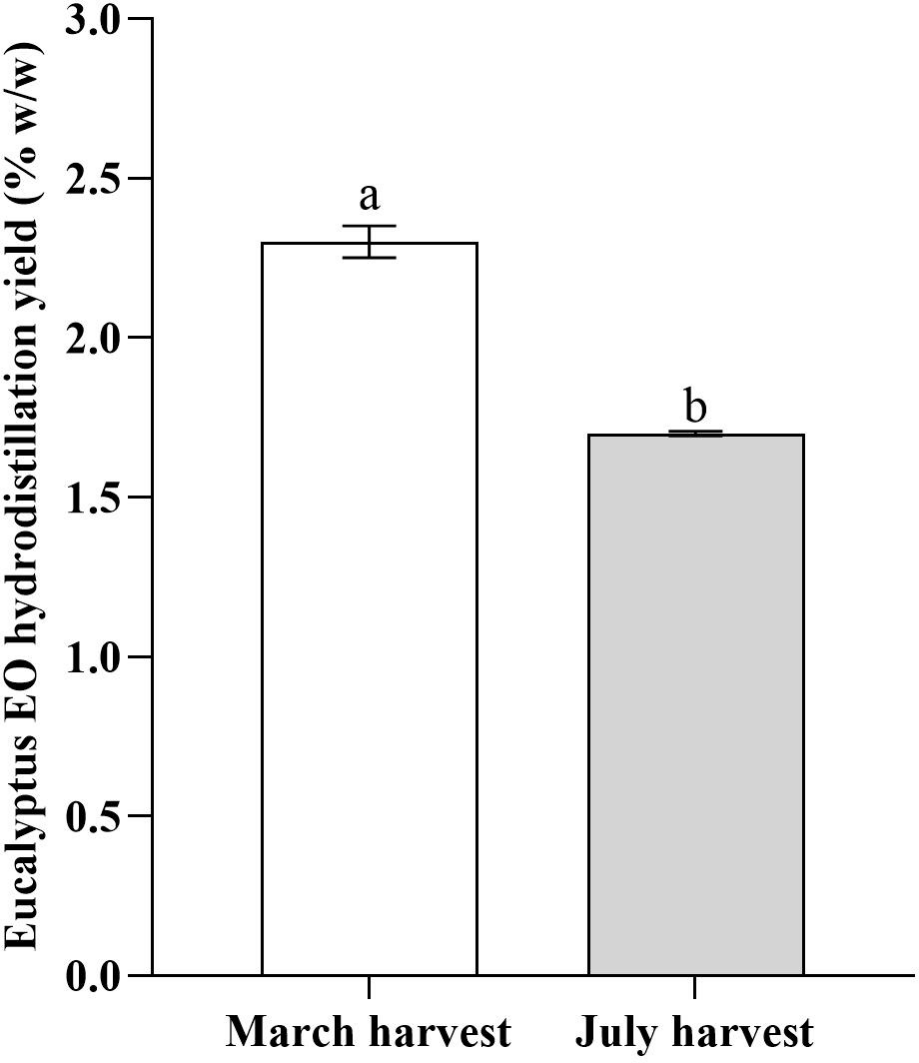
Essential oil hydrodistillation yield (%, w/w) in leaves of *E. parvula* harvested in March and July. Means followed by different letters are statistically different at *p* ≤ 0.05, according to Student’s *t*-test.

Essential oil (EO) yield showed significant differences between plant organs (leaves and fruits) in both 3- and 4-year-old melaleuca plants, as shown by the significant plant age and plant organ interaction (PA x PO) (**Table 1**; **Figure 1**). In eucalyptus, EO yield differed significantly between leaves harvested in March and July, as shown in **Figure 2**. Specifically, in *M. alternifolia*, essential oil production was statistically influenced by plant age and plant organ. Leaf material from 4-year-old plants exhibited the highest EO yield (5.0%) compared to 3-year-old plants (3.1%), representing a substantial age-related increase of 37.8%. Conversely, fruit-derived EO showed an opposite trend with EO yield decreased from 2.63% in 3-year-old plants to 2.06% in 4-year-old plants. Comparing leaves and fruits in 3-year-old plants, the EO yield found in the leaves was higher than that in the fruits, and this was even more evident in 4-year-old plants, with a EO yield in the leaves more than double that in the fruits.

**Table 1:** ANOVA table with F-values and statistical significance for TTEO yield (% w/w) in leaves and fruits derived from 3- and 4-year old melaleuca plants.

Source of variation	TTEO yield (% *w/w*)
Plant age (PA)	77.32**
Plant organ (PO)	149.4***
PA x PO	22.26***

According to LSD test: ** significant at *p* ≤ 0.01; *** significant at *p* ≤ 0.001. Variability factors: Plant age (PA), Plant organ (PO).

Also, EO yield in *E. parvula* was statically influenced by harvesting time with a significant decrease in EO yield observed from early spring to mid-summer. The March harvest yielded 2.3% (*w/w*) EO, whereas leaf material collected in July produced only 1.7% (w/w).

### 3.2 EO composition of *Melaleuca alternifolia*

**Figure 3:**
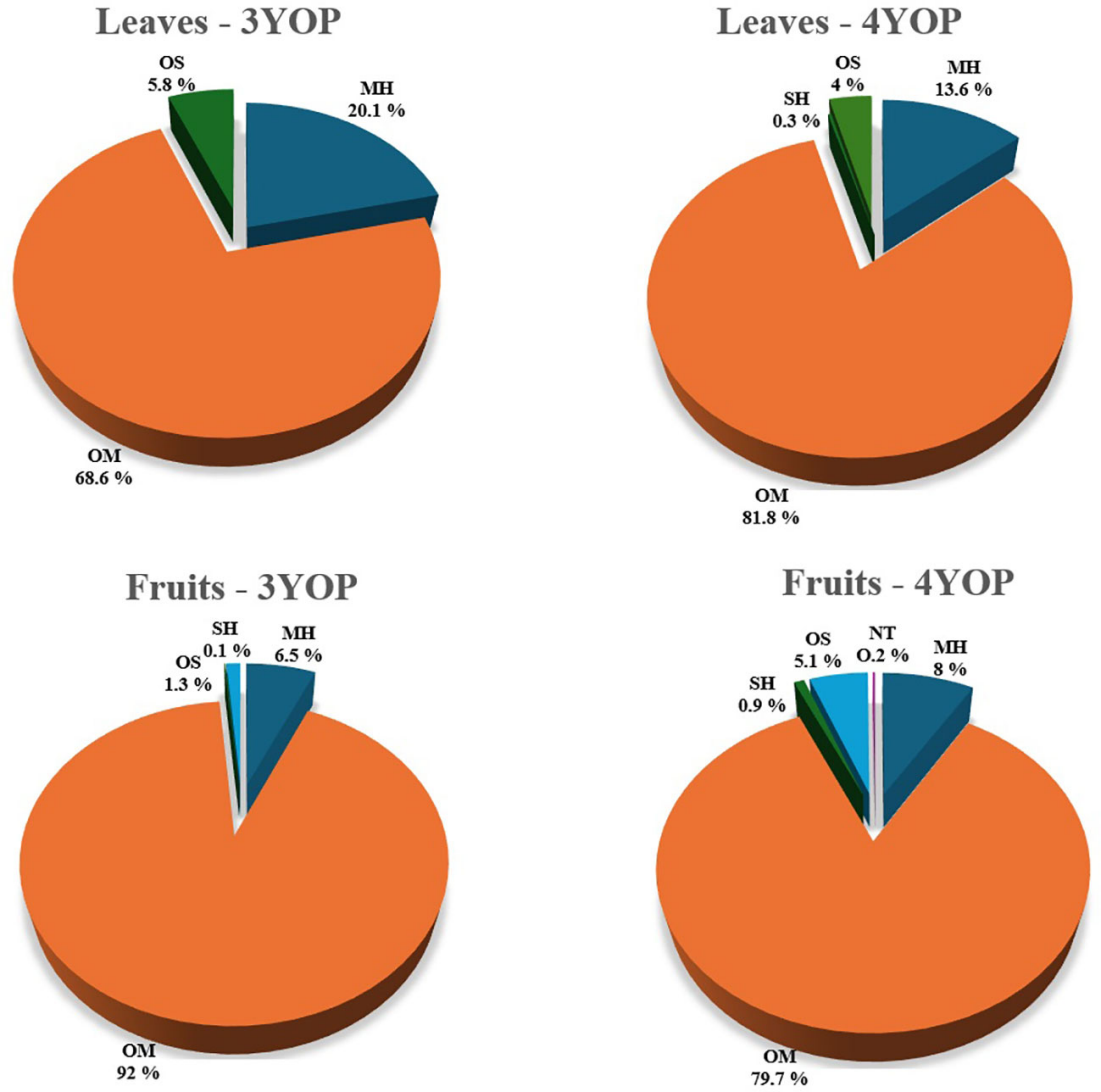
Main chemical classes identified in TTEO composition of leaves and fruits deriving from 3 and 4-year old melaleuca plants. 3YOP = 3-year-old plants; 4YOP = 4-year-old plants. Chemical classes: Monoterpenes hydrocarbons (MH), Oxygenated monoterpenes (OM), Sesquiterpene hydrocarbons (SH), Oxygenated sesquiterpenes (OS).

Tea tree essential oils (TTEOs) obtained from 3-year-old plants by hydrodistillation of leaves and fruits showed clear quali-quantitative differences in their chemical profiles (**Table 2**; **Figure 3**). Overall, in 3-year-old plants, the identified constituents accounted for 94.5% and 99.9% of the total EO in leaves and fruits, respectively, while in 4-year-old plants they accounted for 99.7% in leaves and 93.9% in fruits, respectively.

**Table 2:** TTEO composition of leaves and fruits deriving from 3- and 4-year-old *Melaleuca alternifolia* plants.

*Melaleuca alternifolia*			3-year-old plants		4-year-old plants	
Compounds ^a^	l.r.i ^b^	Class	Leaves (%)	Fruits(%)	Leaves (%)	Fruits (%)
α-thujene	931	MH	- **^c^**	–	tr	0.1 ± 0.001
tricyclene	934	MH	2.3 ± 0.01	–	–	–
α-pinene	939	MH	–	1.3 ± 0.001	2.0 ± 0.01	1.1 ± 0.008
β-pinene *	**980**	**MH**	**0.1 ± 0.001 ^B^**	**0.6 ± 0.003 ^A^**	**0.7 ± 0.002 ^A^**	**0.3 ± 0.001 ^B^**
myrcene *	**992**	**MH**	**1.9 ± 0.05 ^A^**	**0.9 ± 0.002 ^B^**	**1.6 ± 0.05 ^A^**	**0.4 ± 0.002 ^B^**
α-phellandrene	1005	MH	0.5 ± 0.001	–	–	0.1 ± 0.001
α-terpinene *	**1018**	**MH**	**2.4 ± 0.01 ^A^**	**0.7 ± 0.003 ^B^**	**2.5 ± 0.03 ^A^**	**2.0 ± 0.05 ^B^**
*o*-cymene	1022	MH	0.3 ± 0.002	–	–	0.5 ± 0.002
*p*-cymene	1026	MH	–	0.2 ± 0.001	0.4 ± 0.01	–
limonene	1031	MH	tr	tr	tr	tr
1,8-cineole *	**1034**	**OM**	**49.1 ± 0.28 ^B^**	**70.9 ± 0.21 ^A^**	**54.4 ± 0.50 ^A^**	**37.4 ± 0.34 ^B^**
γ-terpinene *	**1062**	**MH**	**4.4 ± 0.35 ^A^**	**1.4 ± 0.02 ^B^**	**5.4 ± 0.03 ^A^**	**3.0 ± 0.02 ^B^**
terpinolene *	**1089**	**MH**	**8.2 ± 0.26 ^A^**	**1.4 ± 0.01 ^B^**	**1.0 ± 0.02 ^A^**	**0.5 ± 0.001 ^B^**
linalool	1100	OM	–	0.2 ± 0.001	–	–
*trans-p*-menth-2,8-dien-1-ol	1125	OM	–	0.3 ± 0.001	–	0.2 ± 0.001
δ-terpineol	1170	OM	tr	7.7 ± 0.01	0.2 ± 0.001	0.2 ± 0.001
terpinen-4-ol *	**1178**	**OM**	**9.5 ± 0.03 ^A^**	**7.7 ± 0.07 ^B^**	**16.5 ± 0.09 ^B^**	**29.0 ± 0.91 ^A^**
α-terpineol *	**1190**	**OM**	**10.0 ± 0.09 ^B^**	**12.9 ± 0.92 ^A^**	**10.7 ± 0.09 ^B^**	**12.9 ± 0.80 ^A^**
α-guaiene	1439	SH	–	–	–	0.2 ± 0.001
viridiflorene	1494	SH	–	–	–	0.2 ± 0.001
δ-cadinene	1524	SH	–	0.1 ± 0.001	0.3 ± 0.001	0.5 ± 0.002
spathulenol	1575	OS	0.4 ± 0.001	0.2 ± 0.001		0.5 ± 0.001
globulol *	**1583**	**OS**	**2.2 ± 0.05 ^A^**	**0.4 ± 0.002 ^B^**	1.4 ± 0.07	1.5 ± 0.05
viridiflorol *	**1590**	**OS**	**2.7 ± 0.02**	–	0.5 ± 0.01	–
guaiol	1596	OS	–	0.3 ± 0.001	0.6 ± 0.001	0.8 ± 0.002
5-*epi*-7-*epi*-α-eudesmol *	**1606**	**OS**	**0.5 ± 0.002 ^B^**	**0.1 ± 0.001 ^A^**	**1.2 ± 0.05 ^A^**	**0.4 ± 0.002 ^B^**
1-*epi*-cubenol	1628	OS	–	0.3 ± 0.002	0.3 ± 0.001	0.9 ± 0.001
cubenol	1641	OS	–	–	–	0.8 ± 0.002
α-cadinol	1654	OS	–	–	–	0.1 ± 0.001
selin-11-en-4-α-ol (=kongol)	1655	OS	–	–	–	0.1 ± 0.009
1-heptadecane	1695	NT	–	–	–	0.2 ± 0.010
**Total identified (%)**			**94.5**	**99.9**	**99.7**	**93.9**

^a^Linear retention index experimentally determined on an HP5-MS capillary column. ^b^Only constituents ≥ 0.1% are reported. ^c^not detected; traces < 0.1%. Compounds reported

in bold were subjected to Student’s *t*-test; different superscript uppercase letters indicate significant differences (*, significant at *p* ≤ 0.05) between melaleuca samples from 3- and 4-year-old plants.

In particular, TTEO obtained from the leaves of 3-year-old plants showed that oxygenated monoterpenes were the predominant chemical class (68.6%), mainly represented by 1,8-cineole (49.1%), α-terpineol (10.0%), and terpinen-4-ol (9.5%). However, a notably high proportion of monoterpene hydrocarbons (20.1%) was also detected, indicating a substantial contribution of the non-oxygenated fraction to the overall oil composition. Within this class, the most representative compounds were terpinolene (8.2%), γ-terpinene (4.4%), and α-terpinene (2.4%). A significant amount of oxygenated sesquiterpenes (5.8%) was also identified, resulting higher than in the TTEO obtained from fruits, suggesting organ-specific metabolic allocation. In contrast, the fruits produced a EO particularly rich in oxygenated monoterpenes (92.0%), but with a distinctly different distribution of the major compounds. The dominant component was 1, 8-cineole occurring at a higher concentration (+44,4%) than in leaves, together with an increase of α-terpineol (+29.0%), while terpinen-4-ol showed a notable decrease (−18.9%). Conversely, monoterpene hydrocarbons significantly decreased (6.5%), particularly terpinolene (-97.6%), γ-terpinene (-81.8%), and α-terpinene (-87.5%). Minor amounts of sesquiterpene hydrocarbons (0.1%) and oxygenated sesquiterpenes (1.3%) were also detected.

The TTEOs obtained from 4-year-old plants also showed quali-quantitative differences depending on leaves or fruits used for hydrodistillation as reported in **Table 2** and **Figure 3**. TTEO obtained from leaves revealed the highest proportion of oxygenated monoterpenes (81.8%), dominated by 1,8-cineole (54.4%), terpinen-4-ol (16.5%), and α-terpineol (10.7%), while monoterpene hydrocarbons accounted for 13.6%, mainly represented by α-terpinene (2.5%), γ-terpinene (5.4%), and terpinolene (1.0%); a small amount of oxygenated sesquiterpenes (4.0%) represented by globulol, viridiflorol, and guaiol and only trace amounts of sesquiterpene hydrocarbons (0.3%) were also detected. Conversely, the fruits produced a EO particularly rich in oxygenated monoterpene compounds (79.7%), with a distinct distribution of the major constituents. While 1, 8-cineole remained the principal compound, its concentration was lower in fruits (-31.3%) than in leaves. In contrast, the fruits substantially accumulated higher amounts of terpinen-4-ol (+43%) and α-terpineol (+17.1%), indicating an accumulation of terpineol isomers in the reproductive organs. Furthermore, monoterpene hydrocarbons were less abundant in fruits (8.0%) with consistent decreases in γ-terpinene (-44.0%), α-terpinene (-20.0%), and terpinolene (- 50%) compared to leaves. Conversely, fruits showed slightly higher levels of oxygenated sesquiterpenes (5.1%) and sesquiterpene hydrocarbons (0.9%), along with non-terpenoid derivatives (0.2%).

### 3.3 EO composition of *E. parvula*

**Figure 4:**
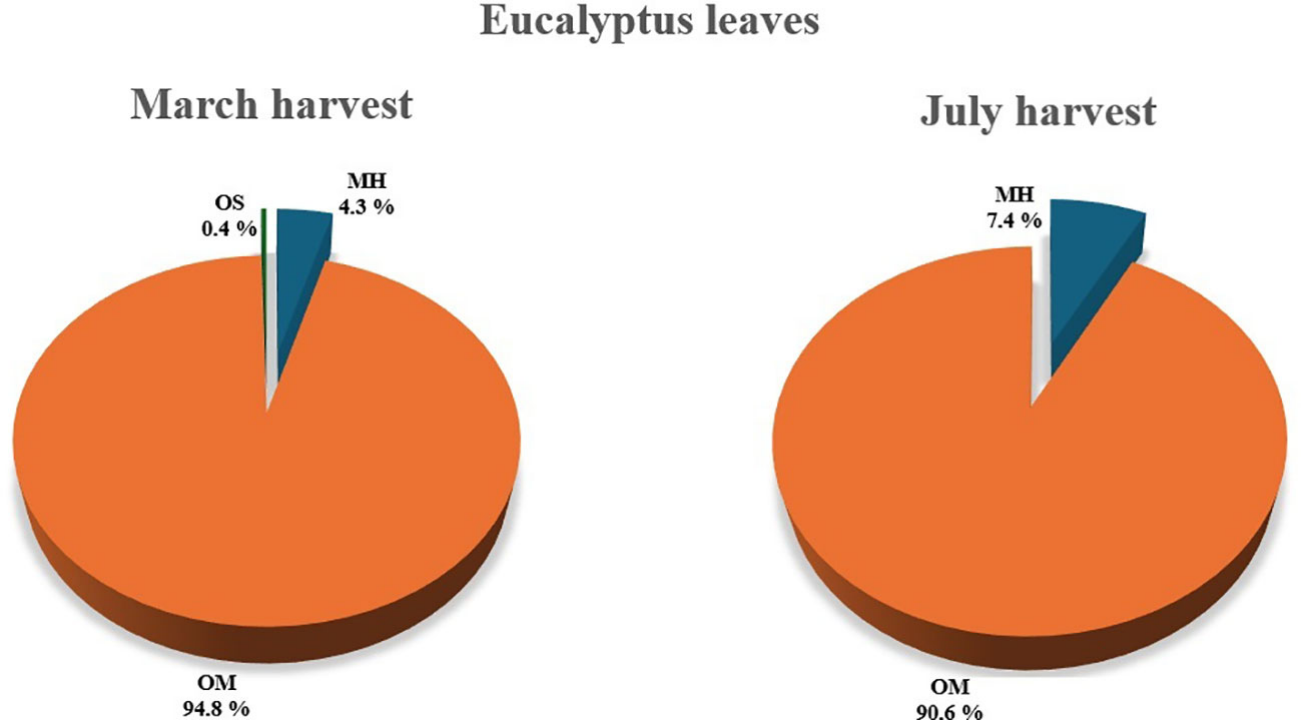
Main chemical classes identified in EO composition of leaves harvested in March and July from 2-year old eucalyptus plants. Chemical classes: Monoterpenes hydrocarbons (MH), Oxygenated monoterpenes (OM), and Oxygenated sesquiterpenes (OS).

The EOs obtained from 2-year-old *E. parvula* leaves harvested in March and July exhibited a characteristic chemical profile dominated by oxygenated monoterpenes, with small percentages of monoterpene hydrocarbons and traces of oxygenated sesquiterpenes (**Figure 4**). Globally, 15 constituents were characterized, accounting for 98.0 to 99.5% of the total oil (**Table 3**).

**Table 3:** EO composition of leaves deriving from March and July harvest of 2-year-old *Eucalyptus parvula* plants.

*2-year-old eucalyptus parvula plants*			Relative Abundance ± Standard Deviation (n=3)	
Compounds ^a^	l.r.i ^b^	Class	Leaves from March harvest	Leaves from July harvest
**α-pinene**	**939**	**MH**	**2.0 ± 0.01**	**2.0 ± 0.01**
myrcene	992	MH	0.2 ± 0.001	tr
α-phellandrene	1005	MH	0.1 ± 0.001	tr
*p*-cymene	1027	MH	0.8 ± 0.005	tr
limonene	1031	MH	-**^c^**	tr
1,8-cineole *	**1034**	**OM**	**90.0 ± 0.26 ^A^**	**82.2 ± 0.45 ^B^**
*(Z)*-β-ocimene *	**1041**	**MH**	**0.7 ± 0.001 ^B^**	**3.2 ± 0.05 ^A^**
i-β-ocimene	1050	MH	0.1 ± 0.001	–
γ-terpinene *	**1062**	**MH**	**0.4 ± 0.001 ^B^**	**2.2 ± 0.07 ^A^**
δ-terpineol	1170	OM	0.1 ± 0.001	–
terpinen-4-ol	1178	OM	0.4 ± 0.002	tr
α-terpineol *	**1190**	**OM**	**3.9 ± 0.04 ^B^**	**6.4 ± 0.03 ^A^**
methyl geranate *	**1325**	**OM**	**0.4 ± 0.001 ^B^**	**2.0 ± 0.001 ^A^**
spathulenol	1575	OS	0.3 ± 0.001	–
viridiflorol	1590	OS	0.1 ± 0.001	–
**Total identified (%)**			**99.5%**	**98.0%**

^a^Linear retention index experimentally determined on an HP5-MS capillary column. ^b^Only constituents ≥ 0.1% are reported. ^c^not detected; traces, < 0.1%. Compounds reported in bold, were subjected to Student’s *t*-test; different superscript uppercase letters indicate significant differences (*, significant at *p* ≤ 0.05) between eucalyptus leaves sampled in March and July, respectively.

The EO obtained from leaves collected in March was almost entirely composed of oxygenated monoterpenes (94.8%). Within this chemical class, 1, 8-cineole represented the most abundant compound, accounting for 90% of the total oil. This dominance was accompanied by relatively minor amounts of α-terpineol, terpinen-4-ol, and other oxygenated derivatives such as δ-terpineol and methylgeranate, all present in low percentages. Monoterpene hydrocarbons modestly contributed to the oil composition (4.3%), mainly represented by α-pinene, p-cymene, and γ-terpinene. Oxygenated sesquiterpenes were negligible (0.4%), primarily represented by spatulenol and viridiflorol.

Similarly, the EO obtained from leaves harvested in July revealed a qualitatively comparable composition, but with evident quantitative variations. Oxygenated monoterpenes still representing the dominant class, though their overall percentage decreased to 90.6%, largely due to a reduction of 1, 8-cineole (-8.6%). In contrast, other oxygenated monoterpenes increased, particularly α-terpineol (+39.06%) and methyl geranate (+80%), compared to the March harvest. Likewise, monoterpene hydrocarbons such as (Z)-β-ocimene (+78.13%) and γ-terpinene (+41.89%) also increased during the summer harvest, suggesting a season-induced modulation of terpene biosynthesis.

### 3.4 VOCs profile of *M. alternifolia*

The volatile organic compounds (VOCs) spontaneously emitted by leaves and fruits of *M. alternifolia* showed marked qualitative and quantitative differences in relation to plant age (**Table 4**). In leaf samples, the VOC profile was consistently dominated by monoterpene hydrocarbons, which accounted for 92.1% and 99.6% of total emissions from 3- and 4-year-old plants, respectively. Within this chemical class, the main constituents were γ-terpinene (16.0–33.2%), 1, 8-cineole (28.8–14.2%), α-terpinene (10.2–20.2%), and terpinolene (27.4% in 3-year-old leaves). Oxygenated monoterpenes (OM) were less abundant in leaves from 3-year-old plants (2.6%) compared to 4-year-old plants (7.7%), primarily due to the rise in terpinen-4-ol (+ 68.9%), a compound typically associated with leaf maturation and oxidative terpene metabolism.

**Table 4:** Spontaneous emissions of VOCs from leaves and fruits derived from melaleuca 3 and 4-year old plants.

Compounds ^a^	l.r.i. ^b^	Class	*Melaleuca alternifolia*					
			3-year-old plants			4-year-old plants		
			Leaves	Fruits		Leaves		Fruits
			Relative Abundance (%)			Relative Abundance (%)		
α-thujene	931	MH	1.2 ± 0.02		-**^c^**	1.6 ± 0.02	0.3 ± 0.001	
α-pinene *	**939**	**MH**	**4.7 ± 0.01 ^A^**		**3.9 ± 0.03 ^B^**	**7.0 ± 0.001 ^A^**	**2.9 ± 0.03 ^B^**	
camphene	953	MH	–		–	–	0.2 ± 0.001	
β-pinene *	**980**	**MH**	**1.4 ± 0.01 ^B^**		**1.9 ± 0.01 ^A^**	**1.9 ± 0.01 ^A^**	**0.6 ± 0.001 ^B^**	
myrcene *	**991**	**MH**	**3.3 ± 0.02 ^B^**		**4.9 ± 0.02 ^A^**	**2.8 ± 0.001 ^A^**	**0.7 ± 0.002 ^B^**	
α-phellandrene *	**1005**	**MH**	**2.3 ± 0.02 ^A^**		**0.5 ± 0.002 ^B^**	**0.7 ± 0.002 ^A^**	**0.2 ± 0.001 ^B^**	
α-terpinene *	**1018**	**MH**	**10.2 ± 0.12 ^A^**		**0.3 ± 0.001 ^B^**	**20.2 ± 0.21 ^A^**	**5.0 ± 0.02 ^B^**	
*p*-cymene *	**1026**	**MH**	**-**		**-**	**3.8 ± 0.02 ^B^**	**6.5 ± 0.02 ^A^**	
1,8-cineole *	**1034**	**MH**	**28.8 ± 0.23 ^B^**		**79.5 ± 0.44 ^A^**	**14.2 ± 0.67 ^A^**	**8.3 ± 0.07 ^B^**	
γ-terpinene *	**1062**	**MH**	**16.0 ± 0.89 ^A^**		**0.8 ± 0.002 ^B^**	**33.2 ± 0.28 ^A^**	**17.4 ± 0.09 ^B^**	
terpinolene *	**1088**	**MH**	**27.4 ± 0.56 ^A^**		**0.2 ± 0.001 ^B^**	**6.1 ± 0.03 ^A^**	**3.2 ± 0.01 ^B^**	
perillene	1099	MH	–		–	–	0.9 ± 0.001	
*trans*-pinocarveol	1140	OM	–		–	–	0.1 ± 0.001	
camphor	1144	OM	–		–	0.1 ± 0.001	–	
terpinen-4-ol *	**1178**	**OM**	**2.3 ± 0.03 ^A^**		**0.5 ± 0.001 ^B^**	**7.4 ± 0.02 ^B^**	**12.3 ± 0.12 ^A^**	
α-terpineol *	**1190**	**OM**	**0.3 ± 0.001 ^B^**		**3.0 ± 0.01 ^A^**	**0.2 ± 0.001 ^B^**	**0.6 ± 0.02 ^A^**	
isobornyl acetate	1286	OM	–		–	–	0.1 ± 0.001	
isoascaridole	1301	OM	–		–	–	0.3 ± 0.001	
δ-elemene	1339	SH	–		–	–	0.2 ± 0.001	
α-cubebene	1351	SH	–		0.2 ± 0.001	–	0.7 ± 0.002	
eugenol	1356	NT	–		–	–	0.3 ± 0.001	
isoledene	1374	SH	–		–	–	0.7 ± 0.001	
α-copaene	1376	SH	–		0.3 ± 0.001	–	1.1 ± 0.005	
β-patchoulene	1380	SH	–		–	–	0.8 ± 0.001	
α-gurjunene *	**1409**	**SH**	**0.1 ± 0.001 ^B^**		**0.5 ± 0.001 ^A^**	**0.1 ± 0.001 ^B^**	**3.7 ± 0.01 ^A^**	
β-caryophyllene	1418	SH			0.4 ± 0.002	–	2.9 ± 0.02	
β-gurjunene	1432	SH	–		–	–	0.2 ± 0.001	
α-guaiene	1438	SH	–		–	0.2 ± 0.001	–	
aromadendrene	1440	SH	0.2 ± 0.001		0.8 ± 0.010	–	7.7 ± 0.03	
α-humulene	1454	SH	–		–	–	0.4 ± 0.001	
α-*neo*-clovene	1456	SH	–		–	–	1.3 ± 0.05	
alloaromadendrene	1461	SH	–		0.5 ± 0.001	0.1 ± 0.001	2.7 ± 0.03	
*cis*-muurola 4(14),5-diene	1463	SH	–		–	–	0.4 ± 0.001	
*trans*-cadina-1(6),4-diene	1470	SH	–		0.2 ± 0.001	–	1.2 ± 0.06	
γ-muurolene	1477	SH	–		–	–	0.2 ± 0.001	
β-selinene	1485	SH	–		–	–	0.3 ± 0.001	
valencene	1490	SH	–		–	–	0.7 ± 0.001	
viridiflorene	1494	SH	–		0.5 ± 0.001	–	2.9 ± 0.03	
bicyclogermacrene	1498	SH	0.2 ± 0.002		–	0.2 ± 0.003	–	
α-muurolene	1500	SH	–		–	–	0.6 ± 0.002	
*(E,E)*-α-farnesene	1508	SH	–		–	–	3.9 ± 0.05	
δ-cadinene *	**1524**	**SH**	**0.1 ± 0.001 ^B^**		**0.9 ± 0.001 ^A^**	**0.1 ± 0.001 ^B^**	**4.3 ± 0.03 ^A^**	
cadina-1,4-diene	1533	SH	–		–	–	0.6	
β-davanone-2-ol	1719	OS	–		–	–	0.8	
Chemical classes								
Monoterpene hydrocarbons (MH)			95.3		92.0	91.5	45.6	
Oxygenated monoterpenes (OM)			2.6		3.5	7.7	13.4	
Sesquiterpene hydrocarbons (SH)			0.6		1.6	0.4	32.0	
Oxygenated sesquiterpenes (OS)			–		–	–	0.8	
Non-terpenes (NT)			–		–	–	0.3	
**Total identified (%)**			**98.5**		**97.1**	**99.6**	**92.1**	

^a^Only constituents ≥ 0.1% are reported; ^b^Linear retention indices (HP-5 column). ^c^not detected; traces < 0.1%. Compounds reported in bold, were subjected to Student’s *t*-test; different superscript uppercase letters indicate significant differences (*, significant at *p* ≤ 0.05) between melaleuca samples from 3- and 4-year-old plants.

In contrast, the VOCs spontaneously released by fruits exhibited a distinctly different chemical composition (**Table 3**). Although fruits from 3-year-old plants were still dominated by monoterpene hydrocarbons (92.0%), fruits from 4-year-old plants showed a marked decrease of them (- 50.4%), associated with a significant increase in sesquiterpene hydrocarbons (SH), which reached 32.0% of the total VOC profile. This change was driven by significant increases in aromadendrene (+89.6%), α-gurjunene (+ 86.5%), viridiflorene (+82.8%), δ-cadinene (+ 79.1%), and the emission of (E, E)-α-farnesene (3.9%) completely absent in fruits of 3-year-old plants. Oxygenated monoterpenes were also increased in fruits of 4-year-old plants (13.4%) compared to 3-year-old ones (3.5%), largely due to 4-terpineol (+95.9%), while oxygenated sesquiterpenes were detected only in fruits of 4-year-old plants, with β-davanone-2-ol (0.8%) as the only representative compound as well as non-terpene derivatives in negligible amounts. Comparing leaves and fruits within the same plant age, organ-specific differences emerged. In 3-year-old melaleuca plants, leaves and fruits shared a VOC profile largely dominated by monoterpene hydrocarbons; however, fruits showed a strong enrichment in 1, 8-cineole (79.5%) compared to leaves (28.8%), followed by a strong reduction in other monoterpene hydrocarbons such as terpinolene and γ-terpinene and a slightly greater contribution of oxygenated monoterpenes. In 4-year-old melaleuca plants, the differences were even more pronounced, with leaves maintaining a VOC profile dominated by monoterpene hydrocarbons, while fruits showed an increase in sesquiterpene hydrocarbons and oxygenated monoterpenes, including aromadendrene, α-gurjunene, δ-cadinene and terpinen-4-ol, highlighting a strong organ-dependent modulation of terpene molecules emission that resulted more evident with advancing plant age.

### 3.5 VOCs profile of *Eucalyptus parvula*

The volatile organic compounds (VOCs) spontaneously emitted by leaves of *Eucalyptus parvula* collected in March and July were characterized through SPME analysis and the results are reported in **Table 5**. In both harvest time, the emission profile was dominated by oxygenated monoterpenes, which accounted for 86.5% of the total VOCs in March and 76.3% in July, respectively, confirming the species characteristic chemotype. Within this class, 1, 8-cineole was by far the most abundant constituent, accounting for 85.9% of emissions in March and 75.4% in July. Monoterpene hydrocarbons were the second most abundant chemical class, increasing from 12.5% ​​in March to 20.1% in July. This seasonal increase was primarily driven by increased emissions of α-pinene (+ 16.2%) and γ-terpinene (+50%), along with moderate increases in myrcene (+ 47.8%), α-phellandrene (+ 53.3%), and allo-ocimene (+ 61.9%). These compounds are often associated with elevated plant metabolic activity under conditions of intense heat and brighter days, suggesting a temperature- and light-dependent modulation of terpene biosynthesis. Other chemical classes showed smaller contributions, such as sesquiterpene hydrocarbons (SH), which increased in July (2.1%) compared to March harvest (0.4%), mainly due to β-caryophyllene and bicyclogermacrene, while non-terpenes content was negligible (0.2–0.4%).

**Table 5:** Spontaneous emissions of VOCs from leaves derived from 2-year-old eucalyptus plants.

			*Eucalyptus parvula leaves*	
Constituents ^a^	LRI ^b^	Class	Relative Abundance (%)	
			(March harvest)	(July harvest)
*(E)*-2-hexenal	854	NT	- **^c^**	0.2 ± 0.001
1-hexanol	867	NT	–	0.2 ± 0.001
α-thujene	931	MH	–	0.1 ± 0.001
α-pinene *	**939**	**MH**	**6.2 ± 0.01 ^B^**	**7.4 ± 0.02 ^A^**
β-pinene	980	MH	0.4 ± 0.001	0.4 ± 0.001
myrcene *	**992**	**MH**	**1.2 ± 0.001 ^B^**	**2.3 ± 0.002 ^A^**
α-phellandrene	1005	MH	0.7 ± 0.001	1.5 ± 0.02
α-terpinene	1018	MH	–	0.6 ± 0.002
*p*-cymene	1027	MH	0.1 ± 0.001	0.1 ± 0.001
limonene	1031	MH	0.2 ± 0.001	–
1,8-cineole *	**1034**	**OM**	**85.9 ± 0.25 ^A^**	**75.4 ± 0.23 ^B^**
*(E)*-β-ocimene	1050	MH	0.3 ± 0.02	–
γ-terpinene *	**1062**	**MH**	**2.6 ± 0.001 ^B^**	**5.2 ± 0.02 ^A^**
terpinolene	1088	MH	–	0.4 ± 0.001
nonanal	1102	NT	–	–
*allo* ocimene *	**1380**	**MH**	**0.8 ± 0.001 ^B^**	**2.1 ± 0.01 ^A^**
terpinen-4-ol *	**1178**	**OM**	**0.2 ± 0.03 ^A^**	**0.1 ± 0.01 ^B^**
**α-terpineol**	**1190**	**OM**	**0.4 ± 0.001 ^A^**	**0.2 ± 0.002 ^B^**
methyl geranate	1324	OM	–	0.6 ± 0.01
δ-elemene	1340	SH	–	0.3 ± 0.002
β-caryophyllene *	**1418**	**SH**	**0.2 ± 0.001 ^B^**	**1.1 ± 0.05 ^A^**
bicyclogermacrene *	**1494**	**SH**	**0.2 ± 0.002 ^B^**	**0.7 ± 0.001 ^A^**
*n*-heptadecane	1700	NT	0.2 ± 0.001	–
Chemical classes				
Monoterpene hydrocarbons (MH)			12.5	20.1
Oxygenated monoterpenes (OM)			86.5	76.3
Sesquiterpene hydrocarbons (SH)			0.4	2.1
Non-terpenes (NT)			0.2	0.4
**Total identified (%)**			**99.6%**	**98.9%**

^a^Only constituents ≥ 0.1% are reported; ^b^Linear retention indices (HP-5 column). ^c^not detected. Compounds reported in bold, were subjected to Student’s *t*-test; different superscript uppercase letters indicate significant differences (*, significant at *p* ≤ 0.05) between eucalyptus leaves sampled in March and July, respectively.

### 3.6 PCA and HCA on *E. parvula* and *M. alternifolia* EOs

**Figure 5:**
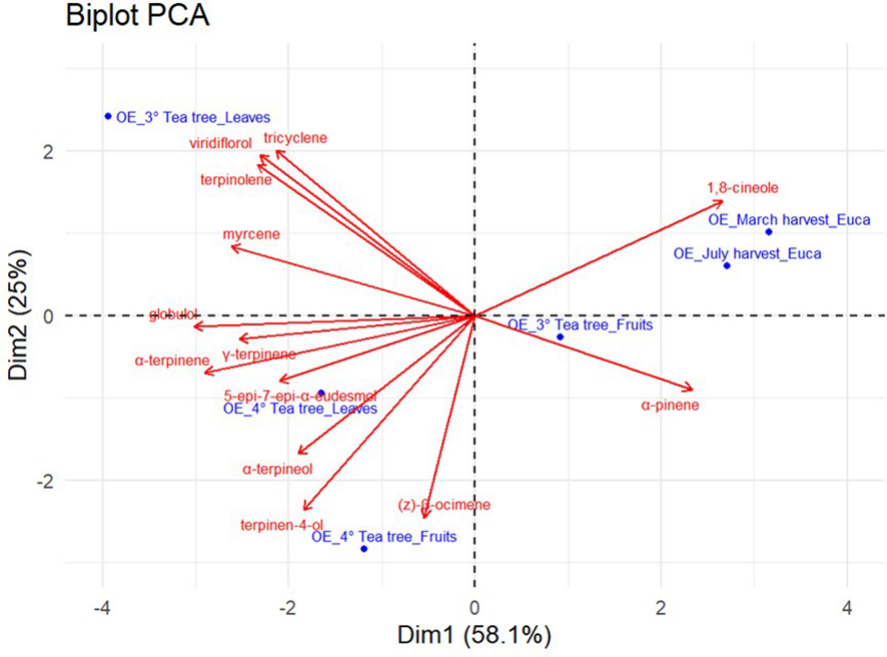
PCA biplot (score plot + loading plot) based on Dim1 (PC1) and Dim2 (PC2) describing the chemodiversity of essential oils among *M. alternifolia* and *E. parvula* samples.

In order to better visualize the variability of chemical profiles of *E. parvula* and *M. alternifolia* EOs and distinguish the effects of plant age, plant organ, and harvest time, a PCA was carried out. The resulting biplot (**Figure 5**) explained 83.1% of the total variance, with PC1 (Dim1) accounting for 58.1% and PC2 (Dim2) accounting for 25.0% of the variability.

PC1 primarily discriminated samples according to the relative abundance of 1, 8-cineole and α-pinene, which showed strong positive loadings along this axis and were mainly associated with *E. parvula* essential oils deriving from leaves collected in March and July. In contrast, negative PC1 scores were associated with viridiflorol, tricyclene, terpinolene, and myrcene, clustering leaf essential oil from 3-year-old tea tree plants. Further variability among tea tree samples was explained along PC2, reflecting differences in plant organ and plant age, with PC2 scores associated with (Z)-β-ocimene, terpinen-4-ol, α-terpineol, 5-epi-7-epi-α-eudesmol, γ-terpinene, and α-terpinene, reflecting organ-specific metabolic variation.

**Figure 6:**
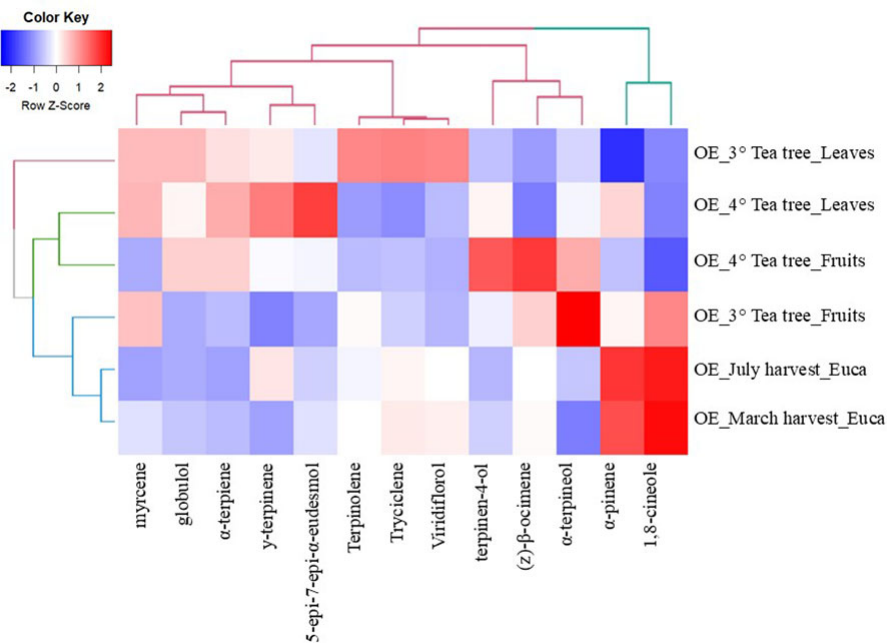
Hierarchical cluster heatmap of the chemical composition of EOs from Tea tree (leaves and fruits of 3- and 4-year-old plants) and Eucalyptus collected at different times (March and July, respectively). Data values were transformed to color scale.

These observations were confirmed by the two-way dendrogram (HCA) reported in **Figure 6**. The clustered heatmap allowed the simultaneous visualization of sample groupings (essential oils from E*. parvula* and *M. alternifolia*, collected from different organs and harvest time) and chemical variables.

HCA revealed a clear separation of the samples into distinct clusters, which largely reflected plant age, organ type, and harvest time. *E. parvula* EOs, deriving from leaves collected in March and July, represented a distinct cluster characterized by a strong dominance of 1,8-cineole and α-pinene, along with the EO derived from the fruits of 3-year-old tea tree plants, which showed a high/intermediate content of 1,8-cineole, α-terpineol, and myrcene. In contrast, tea tree EOs clustered into two main subclusters corresponding to samples derived from leaves and fruits, reflecting organ-specific metabolic differentiation. Furthermore, HCA identified two main groups of chemical compounds that reflected shared biosynthetic pathways and accumulation patterns. The first cluster is dominated by 1,8-cineole and α-pinene, while the second was represented by the remaining terpene compounds, including monoterpene hydrocarbons, oxygenated monoterpenes, and sesquiterpenes. This group is most strongly associated with the essential oils of *M. alternifolia* and reflects a more complex terpene biosynthetic profile, typical of tea tree chemotypes.

### 3.7 Phytotoxicity evaluation

**Figure 7:**
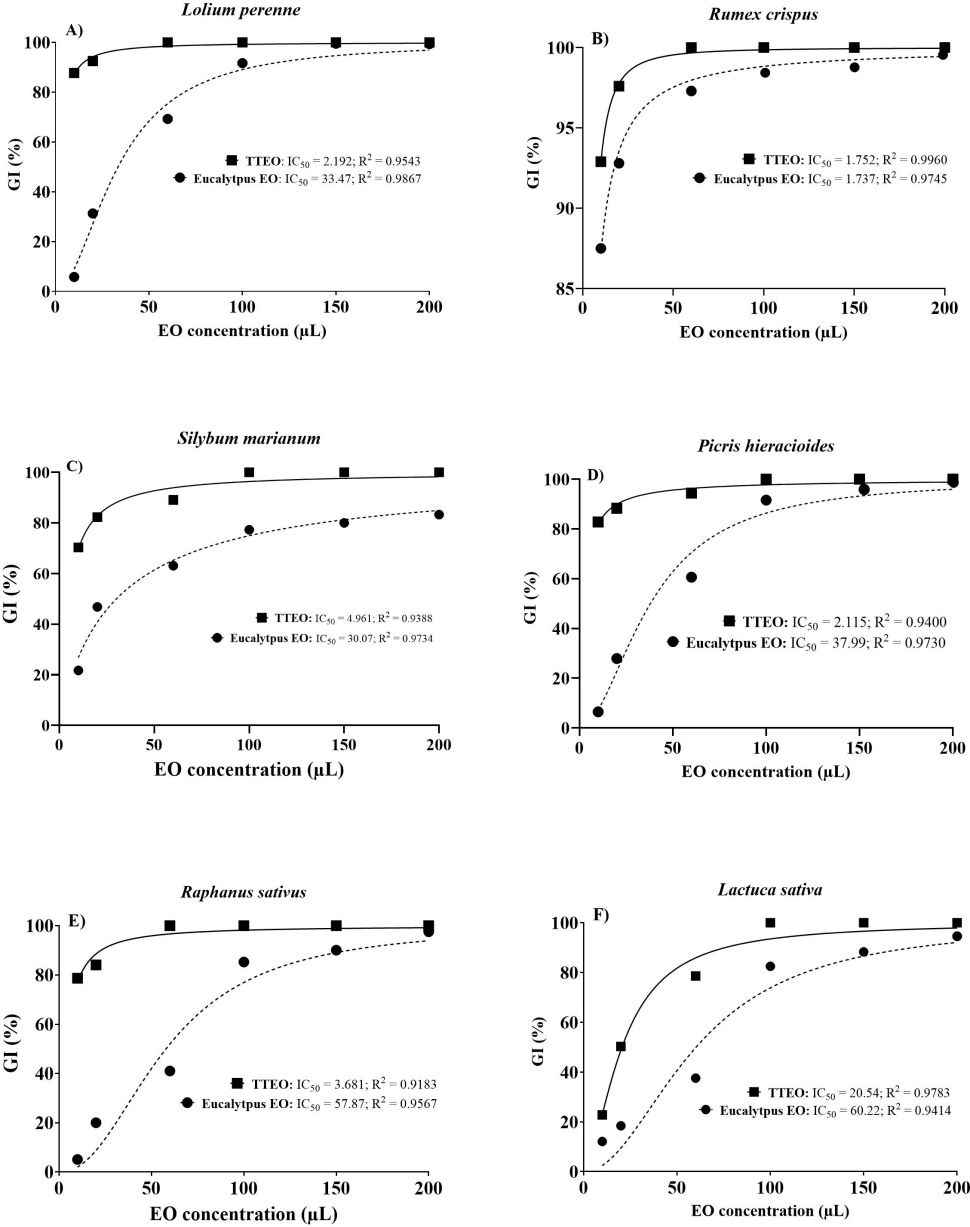
Dose–response curves of seed germination inhibition (GI, %) induced by tea tree essential oil (TTEO) and eucalyptus essential oil (EO) in **(A)***Lolium perenne*, **(B)***Rumex crispus*, **(C)***Silybum marianum*, **(D)***Picris hieracioides*, **(E)***Raphanus sativus*, and **(F)***Lactuca sativa.* Germination percentage inhibition is expressed as a function of increasing essential oil concentration (µL). Solid lines represent TTEO, while dashed lines represent eucalyptus EO. Points indicate mean values, and non-linear regression curves were used to estimate IC_50_ values. Corresponding IC_50_ and R² values are reported within each panel.

**Figure 8:**
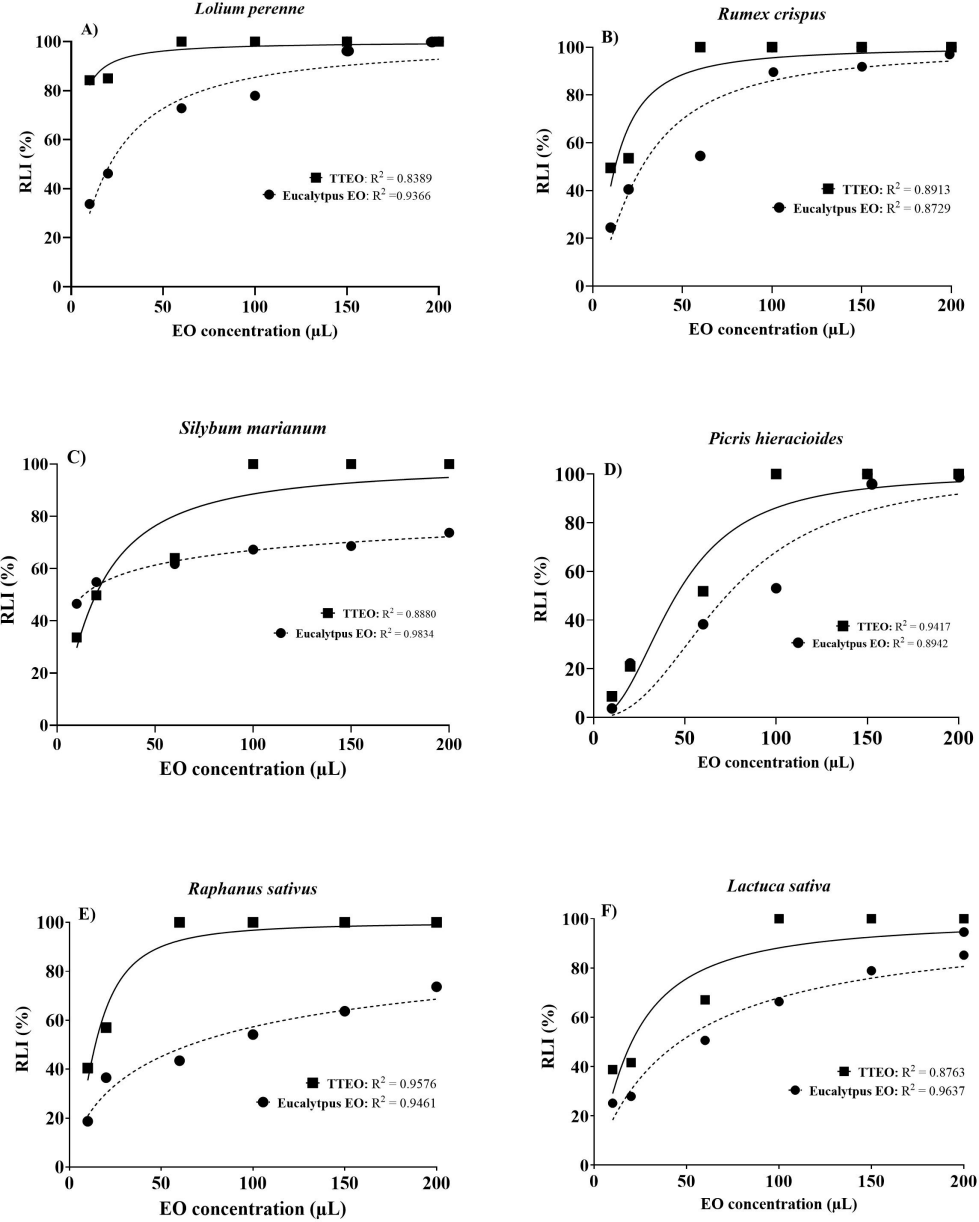
Dose–response curves of radicle length inhibition (RLI %) induced by tea tree essential oil (TTEO) and eucalyptus essential oil (EO) in **(A)***Lolium perenne*, **(B)***Rumex crispus*, **(C)***Silybum marianum*, **(D)***Picris hieracioides*, **(E)***Raphanus sativus*, and **(F)***Lactuca sativa.* Germination percentage inhibition is expressed as a function of increasing essential oil concentration (µL). Solid lines represent TTEO, while dashed lines represent eucalyptus EO. Points indicate mean values, and non-linear regression curves were used to estimate IC_50_ values. Corresponding IC_50_ and R² values are reported within each panel.

Seed germination percentage, radicle length, and mean germination time (MGT) were significantly affected by essential oil type and concentrations in all species tested (**Table 6**). For each species, increasing doses of eucalyptus and tea tree EOs resulted in a statistically significant reduction in seed germination percentage and radicle length compared to the control. TTEO caused stronger and earlier inhibition than eucalyptus EO, leading to a complete suppression of seed germination and radicle length at low concentrations in most species. Conversely, eucalyptus EO showed a gradual and significant dose-dependent effect. MGT was also significant affected by essential oil treatments, with delayed seed germination percentage observed at increasing EO concentrations, particularly at intermediate/high doses (60–100 µL) depending on species and EO type used. These significant differences between treatments are consistent with dose-response curves reported in **Figures 7**, **8**. In particular, **Figure 7** showed the phytotoxic effects of eucalyptus and tea tree EOs on seed germination inhibition in the tested weed species, as well as in *Lactuca sativa* and *Raphanus sativus* used as reference species. In all species, the percentage of seed germination inhibition (GI%) exhibited a clear dose–response relationship for both EOs, with progressively increasing percentages of germination inhibition as the EO concentration increased from 10 to 200 µL. Overall, TTEO showed stronger phytotoxic activity than eucalyptus EO, as indicated by the lower IC_50_ values ​​and higher percentages of inhibition at lower doses. In *L. perenne* (**Figure 7A**) and *P. hieracioides* (**Figure 7D**), TTEO caused a high percentage of germination inhibition at the lowest doses (20 and 60 µL), while eucalyptus EO showed a more gradual inhibition trend, reaching comparable germination inhibition percentage only at higher concentrations. The IC_50_ of TTEO against *L. perenne* was lower (=2.192 µL) compared to IC_50_ of eucalyptus EO (= 33.47 µL), indicating a significantly higher sensitivity to TTEO. A similar pattern was observed in *P. hieracioides*, where TTEO showed a very low IC_50_ = 2.115 µL, whereas for eucalyptus EO is required 37.99 µL to reach the same inhibitory effect. *R. crispus* (**Figure 7B**) proved highly sensitive to both EOs, with very high percentage of germination inhibition values even at low doses, showing very low IC_50_ for both treatments (i.e., TTEO = 1.752; eucalyptus EO = 1.737 µL). Also *S. marianum* (**Figure 7C**) showed greatly higher sensitivity to TTEO, which exhibited a very low IC_50_ value (= 4.961 µL), whereas eucalyptus EO required substantially higher EO concentrations to reach 50% inhibition (IC_50_ = 30.07 µL). A similar trend, to that observed in the weed species, was recorded in *R. sativus* (**Figure 7E**), where TTEO showed a much lower IC_50_ (= 3.681 µL) compared with eucalyptus EO (= 57.87 µL). In contrast, *L. sativa* (**Figure 7F**) was the least sensitive species, showing the highest IC_50_ values for both essential oils (TTEO = 20.54 µL; eucalyptus EO = 60.22 µL). Finally, at the highest concentrations tested (150–200 µL), both EOs caused almost complete germination inhibition in most species.

**Table 6:** Effect of eucalyptus EO and TTEO on seed germination (G %), radicle length (cm), and on mean germination time (MGT, days) of weeds (i.e., *Lolium perenne*, *Rumex crispus*, *Silybum marianum*, and *Picris hieracioides*) and reference species (i.e*. Raphanus sativus* and *Lactuca sativa*).

Species	Treatment	EO Level (µL)	G (%)	Radicle length (cm)	MGT (days)
*Lolium perenne*	Control	0	84.3 ± 4.8 a	3.94 ± 0.27 a	4.8 ± 0.3 a
	Eucalyptus EO	10	79.1 ± 2.2 a	2.61 ± 0.18 b	5.0 ± 0.2 a
		20	58.2 ± 1.8 b	2.12 ± 0.09 c	5.0 ± 0.1 a
		60	26.3 ± 1.3 c	1.07 ± 0.04 d	6.0 ± 0.1 b
		100	6.0 ± 0.1 d	0.87 ± 0.01 e	6.0 ± 0.1 b
		150	0	0	0
		200	0	0	0
	Control	0	84.3 ± 4.8 a	3.94 ± 0.27 a	4.8 ± 0.3 a
	TTEO	10	10.4 ± 1.1 b	0.62 ± 0.02 b	5.0 ± 0.1 a
		20	5.2 ± 0.6 c	0.59 ± 0.01 b	5.0 ± 0.1 a
		60	0	0	0
		100	0	0	0
		150	0	0	0
		200	0	0	0
*Rumex crispus*	Control	0	84.5 ± 4.1 a	2.00 ± 0.13 a	4.6 ± 0.3 a
	Eucalyptus EO	10	10.6 ± 0.8 b	1.51 ± 0.09 b	5.0 ± 0.1 a
		20	6.1 ± 0.8 c	1.19 ± 0.06 c	5.0 ± 0.1 a
		60	2.3 ± 0.6 d	0.91 ± 0.02 d	6.0 ± 0.1 b
		100	0	0	0
		150	0	0	0
		200	0	0	0
	Control	0	84.5 ± 4.1 a	2.00 ± 0.13 a	4.6 ± 0.3 a
	TTEO	10	6.0 ± 0.1 b	1.01 ± 0.08 b	5.0 ± 0.1 a
		20	2.0 ± 0.01 c	0.93 ± 0.02 b	5.0 ± 0.1 a
		60	0	0	0
		100	0	0	0
		150	0	0	0
		200	0	0	0
*Silybum marianum*	Control	0	89.5 ± 7.9 a	2.17 ± 0.19 a	5.0 ± 0.1 a
	Eucalyptus EO	10	71.2 ± 1.9 b	1.16 ± 0.12 b	5.0 ± 0.1 a
		20	50.0 ± 1.3 c	0.98 ± 0.02 c	5.0 ± 0.1 a
		60	36.2 ± 1.0 d	0.83 ± 0.01 d	6.0 ± 0.1 b
		100	24.2 ± 0.9 e	0.71 ± 0.02 de	6.0 ± 0.1 b
		150	18.3 ± 1.3 f	0.68 ± 0.04 e	6.0 ± 0.1 b
		200	15.0 ± 1.0 f	0.57 ± 0.05 f	6.0 ± 0.1 b
	Control	0	89.5 ± 7.9 a	2.17 ± 0.19 a	5.0 ± 0.1 a
	TTEO	10	30.1 ± 1.1 b	1.44 ± 0.18 b	5.0 ± 0.1 a
		20	20.0 ± 0.2 c	1.09 ± 0.09 c	5.0 ± 0.1 a
		60	14.2 ± 0.9 d	0.78 ± 0.01 d	6.0 ± 0.1 b
		100	0	0	0
		150	0	0	0
		200	0	0	0
*Picris hieracioides*	Control	0	71.0 ± 2.9 a	0.81 ± 0.10 a	5.0 ± 0.1 a
	Eucalyptus EO	10	66.4 ± 1.9 b	0.78 ± 0.04 a	5.0 ± 0.1 a
		20	51.2 ± 2.1 c	0.63 ± 0.01 b	5.0 ± 0.1 a
		60	28.1 ± 1.7 d	0.50 ± 0.02 c	6.0 ± 0.2 b
		100	6.0 ± 0.09 e	0.38 ± 0.01 d	6.0 ± 0.1 b
		150	0	0	0
		200	0	0	0
	Control	0	71.0 ± 2.9 a	0.81 ± 0.10 a	5.0 ± 0.1 a
	TTEO	10	12.2 ± 0.9 b	0.74 ± 0.01 ab	5.0 ± 0.1 a
		20	8.3 ± 0.6 c	0.64 ± 0.03 b	5.0 ± 0.1 a
		60	4.0 ± 0.02 d	0.39 ± 0.02 c	6.0 ± 0.1 b
		100	0	0	0
		150	0	0	0
		200	0	0	0
*Raphanus sativus*	Control	0	95.3 ± 1.5 a	4.60 ± 0.36 a	2.0 ± 0.1 a
	Eucalyptus EO	10	90.4 ± 1.1 b	3.74 ± 0.28 b	2.0 ± 0.1 a
		20	76.2 ± 2.4 c	2.92 ± 0.19 c	2.0 ± 0.1 a
		60	56.2 ± 1.1 d	2.60 ± 0.09 c	2.0 ± 0.1 a
		100	14.0 ± 0.02 e	2.11 ± 0.12 d	3.0 ± 0.2 b
		150	10.2 ± 1.2 f	1.67 ± 0.09 e	3.0 ± 0.1 b
		200	2.3 ± 0.6 g	1.21 ± 0.10 f	3.0 ± 0.1 b
	Control	0	95.3 ± 1.5 a	4.60 ± 0.36 a	2.0 ± 0.1 a
	TTEO	10	20.3 ± 0.2 b	2.74 ± 0.01 b	2.0 ± 0.1 a
		20	15.2 ± 1.0 c	1.98 ± 0.18 c	2.0 ± 0.1 a
		60	0	0	0
		100	0	0	0
		150	0	0	0
		200	0	0	0
*Lactuca sativa*	Control	0	93.3 ± 1.5 a	5.41 ± 0.36 a	2.0 ± 0.1 a
	Eucalyptus EO	10	82.0 ± 1.1 b	4.05 ± 0.28 b	2.0 ± 0.1 a
		20	76.1 ± 1.9 c	3.90 ± 0.17 b	2.0 ± 0.1 a
		60	58.2 ± 0.3 d	2.67 ± 0.18 c	3.0 ± 0.2 b
		100	16.3 ± 1.2 e	1.82 ± 0.11 d	3.0 ± 0.1 b
		150	10.5 ± 1.3 f	1.14 ± 0.09 e	3.0 ± 0.1 b
		200	5.0 ± 1.0 g	0.80 ± 0.10 f	3.0 ± 0.1 b
	Control	0	93.3 ± 1.5 a	5.41 ± 0.36 a	2.0 ± 0.1 a
	TTEO	10	72.0 ± 1.1 b	3.31 ± 0.10 b	2.0 ± 0.1 a
		20	46.4 ± 1.8 c	3.16 ± 0.09 b	2.0 ± 0.1 a
		60	20.0 ± 1.5 d	1.78 ± 0.01 c	3.0 ± 0.1 b
		100	0	0	0
		150	0	0	0
		200	0	0	0

One-way ANOVA was used to evaluate the effects of different EO concentration (10, 20, 60, 100, 150, and 200 µL) of tea tree and eucalyptus on each tested species, in comparison with the untreated control. Values (means ± sd) followed by different letters are statistically different at *p* ≤ 0.05 based on the Least Significant Difference (LSD) test.

A similar dose–response relationship was also observed for radicle length inhibition percentage in all tested species (**Figure 8**). The percentage of radicle length inhibition increased progressively with rising EO concentrations, from 10 to 200 µL in all species, demonstrating a consistent phytotoxic effects also on early seedling development. Generally, TTEO showed higher percentages of radicle length inhibition than eucalyptus essential oil at all EO concentrations, particularly at 20 and 60 µL. In *L. perenne* (**Figure 8A**), TTEO caused a rapid increase in radicle length inhibition, exceeding 80% at low concentration, while eucalyptus EO showed a more gradual dose-dependent increase.

*R. crispus* (**Figure 8B**) appeared highly sensitive to both essential oils, although TTEO induced greatly higher radicle length inhibition percentages at low and intermediate concentrations compared with eucalyptus EO. Conversely, in *S. marianum* (**Figure 8C**), eucalyptus EO produced a more moderate inhibition of radicle length across the whole concentration range, with inhibition values remaining clearly lower than those obtained with TTEO. In *P. hieracioides* (**Figure 8D**), the percentage inhibition of radicle length increased with EO concentration for both treatments, resulting in a complete inhibition at the highest EO concentrations. Regarding reference species, in *R. sativus* (**Figure 8E**), TTEO induced a rapid and almost complete inhibition of radicle elongation at intermediate concentrations, while eucalyptus EO showed a slower and more partial inhibitory effect. In *L. sativa* (**Figure 8F**), both EOs produced progressively higher inhibition values with increasing EO concentration; although TTEO was more effective, the difference between treatments was less pronounced than that observed in *R. sativus*, with eucalyptus EO reaching high inhibition levels at the highest EO concentrations.

## 4 Discussion

In recent years, growing awareness of the environmental impact related to the intensive use of synthetic pesticides has led to renewed interest in plant-based products, which are more easily degradable, pose less risk to non-target organisms, and are potentially effective in countering the pest resistance to conventional chemicals (Gupta et al., 2023; Raveau et al., 2020). In this context, the local cultivation of Australian species such as *E. parvula* and *M. alternifolia* represents a strategic opportunity to create production chains in Italy in which all stages, from the selection of plant material to harvesting, extraction, and product standardization, can be effectively controlled, ensuring high quality, traceability, and environmental sustainability. Our results showed that the chemical profiles of EOs and VOC emissions varied according to plant organ, plant age, and harvest time, supporting the view that EO and VOC composition is not a fixed qualitative trait but the outcome of genotype expression modulated by developmental and environmental factors (Dhifi et al., 2016; Fotsing and Kezetas, 2020; Homer et al., 2000; Tholl, 2006). This variability reflects the dynamic nature of terpene biosynthesis, in which enzymatic activity, precursor availability, and tissue differentiation collectively determine the accumulation of specific chemical compounds (Cheng et al., 2025).

In *M. alternifolia*, previous studies have demonstrated a clear relationship between leaf development stage and EO composition, with younger leaves typically enriched in monoterpene hydrocarbons and mature leaves characterized by a higher proportion of oxygenated monoterpenes (Homer et al., 2000; Webb et al., 2013; Padovan et al., 2017). A similar trend was also observed in our 3- and 4-year-old plants, in which increasing plant age was consistently associated with a greater abundance of oxygenated monoterpenes. This shift likely reflects progressive oxidative modifications of monoterpene hydrocarbons during leaf maturation, resulting in the accumulation of compounds such as terpinen-4-ol and α-terpineol (Bratati, 2023; Mau and Croteau, 2006).

The importance of regulatory mechanisms in influencing EO composition has been highlighted by Webb et al. (2013), who showed that transcriptional regulation of the MEP pathway explains a large proportion of the variation in monoterpene content in *M. alternifolia*. In addition, environmental factors, including temperature, light, water availability, and biotic stress, are known to influence terpene biosynthesis by modulating gene expression and precursor fluxes (Das and Prakash, 2024). Consistent with these observations, our data showed significant differences between leaves and fruits of 3- and 4-year-old *M. alternifolia* plants, as well as between *E. parvula* leaf samples collected in March and July, confirming that EO composition varied in response to both developmental stage and seasonal conditions. In particular, fruits from older *M. alternifolia* plants showed compositional shifts from profiles dominated by 1, 8-cineole toward mixtures enriched in terpinen-4-ol and sesquiterpenes, suggesting a functional reorganization of terpene biosynthesis in reproductive organs. Such changes are consistent with the hypothesis that reproductive organs emitted VOCs in response to specific ecological needs, such as seed protection, defense against pathogens, deterrence of herbivores, or regulation of biotic interactions during the reproductive phase (Bergman et al., 2025; Lo et al., 2024; Mobarak et al., 2025; Russo et al., 2022).

Multivariate analyses (PCA and HCA) further demonstrated that EO chemodiversity was mainly driven by plant developmental stage and harvest time and depended on a limited number of dominant terpene compounds. Samples clustered according to species, plant organ, and harvest period, with *E. parvula* EOs strongly associated with 1,8-cineole and α-pinene, and *M. alternifolia* EOs distributed according to plant age and organ and correlated with several monoterpene hydrocarbons and oxygenated monoterpenes, including terpinen-4-ol, α-terpineol, γ-terpinene, and terpinolene.

VOC emissions also varied with plant age, particularly in reproductive organs. Fruits from 3-year-old plants predominantly emitted monoterpenes, whereas fruits from 4-year-old plants showed an increased relative abundance of sesquiterpenes, confirming the role of reproductive organs as dynamic sources of chemical signals whose composition changes during plant development (Bergman et al., 2025; Chen and Liao, 2025). Accordingly, plant organ and plant age emerged as key determinants of the biological potential of tea tree essential oils (TTEOs), influencing both chemical composition and functional properties.

Regarding *E. parvula*, EO composition was characterized by a high degree of chemotypic stability, with 1, 8-cineole representing the dominant compound at both harvest times. However, EO yield decreased from March to July, and seasonal differences in minor components were observed, with July samples showing increased proportions of hydrocarbon monoterpenes. These changes likely reflect typical Mediterranean summer conditions, which favor monoterpene biosynthesis and volatilization without altering the dominant chemotype (Bulama Modu et al., 2025; De Brito-MaChado et al., 2022; Silva-Pando and Pino-Pérez, 2016). Similar seasonal effects have been reported for other *Eucalyptus* species, indicating a combined influence of leaf maturity and environmental conditions on monoterpene production (Rajapaksha et al., 2023).

Both *M. alternifolia* and *E. parvula* EOs exhibited phytotoxic activity, in agreement with previous evidence that essential oils from Myrtaceae and other aromatic plants can inhibit seed germination and early seedling growth. Essential oils rich in oxygenated monoterpenes, such as 1,8-cineole, terpinen-4-ol, γ-terpinene, and α-terpineol, are known to interfere with key physiological processes, including cell division and membrane integrity (Abd-ElGawad et al., 2020; Khammassi et al., 2022; Verdeguer et al., 2020). Although both essential oils showed strong inhibitory effects, species-specific differences in sensitivity were evident, as reflected by distinct IC_50_ values. Tea tree essential oil consistently exhibited greater phytotoxic potency than eucalyptus EO, inducing higher levels of inhibition at lower concentrations. Overall, phytotoxic responses followed a clear dose-dependent pattern, with increasing inhibition of seed germination and radicle elongation as EO concentration increased. Nevertheless, further studies are required to elucidate the contribution and potential synergistic interactions of individual compounds. Such information will be essential to assess the practical feasibility of using these essential oils as natural herbicides within sustainable weed management strategies.

## 5 Conclusions

Our study indicates that *M. alternifolia* and *E. parvula* can be successfully cultivated under Mediterranean conditions for the production of terpene-rich high-quality essential oils with a great phytotoxic potential. The observed variability, dependent on plant age, organ, and harvest time, highlighted the chemical diversity of these species when grown outside their native range and supports their potential as sources of bioactive compounds. Therefore, these results strengthen the interest in plant-derived essential oils as natural alternatives to synthetic pesticides, with potential applications in agriculture, particularly for the development of bioherbicide formulations, but also in the cosmetic and pharmaceutical sectors. To translate this potential into field−relevant applications and to better connect phytochemical diversity with agroecosystem outcomes, future work should investigate the contribution and possible synergistic interactions of individual compounds, as well as formulation and delivery strategies, and assessments of selectivity and non−target effects (including crops, beneficial organisms, and soil biota). Furthermore, the development of local production chains could contribute to reducing the environmental impact associated with the importation of EOs from non-European countries, while promoting more sustainable local agricultural systems.

## Data Availability

The original contributions presented in the study are included in the article/supplementary material. Further inquiries can be directed to the corresponding author.
